# Long-term crude probabilities of death among breast cancer patients by age and stage: a population-based survival study in Northeastern Spain (Girona–Tarragona 1985–2004)

**DOI:** 10.1007/s12094-018-1852-1

**Published:** 2018-03-06

**Authors:** R. Clèries, A. Ameijide, M. Buxó, J. M. Martínez, R. Marcos-Gragera, M.-L. Vilardell, M. Carulla, Y. Yasui, M. Vilardell, J. A. Espinàs, J. M. Borràs, J. Galceran, À. Izquierdo

**Affiliations:** 1 Pla Director d’Oncologia (GENCAT), IDIBELL, Hospital Duran i Reynals, Gran Via 199-203 1ª planta, L’Hospitalet de Llobregat, 08908 Barcelona, Spain; 2 Departament de Ciències Clíniques, Universitat de Barcelona, Campus de Bellvitge, L’Hospitalet de Llobregat, Barcelona, Spain; 3 Registre de Càncer de Tarragona, Fundació Lliga per a la Investigació i Prevenció del Càncer (FUNCA)-IISPV, Reus, Tarragona Spain; 4 Institut d’Investigació Biomèdica de Girona, IDIBGI, C/Dr.Castany s/n, Edifici M2, Parc Hospitalari Martí i Julià, 17190 Salt, Spain; 5 MC MUTUAL, Departamento de Investigación y Análisis de Prestaciones, C/Provenza, 321, 08037 Barcelona, Spain; 6 Unitat d’Epidemiologia i Registre del Càncer de Girona (UERGG), Institut d’Investigació Biomèdica Girona Josep Trueta (IDIBGI), Girona, Spain; 7 Institut Català d’Oncologia (ICO), Girona, Spain; 8 Departament d’Infermeria, Universitat de Girona (UdG), Girona, Spain; 9 Department of Epidemiology and Cancer Control, St. Jude Children’s Research Hospital, Memphis, TN 38105 USA; 10 Sección de Estadística del Departamento de Genética, Microbiología y Estadística de la Facultad de Biología, Universidad de Barcelona, 08028 Barcelona, Spain; 11 Departament de Medicina i Cirurgia, Universitat Rovira i Virgili, Reus, Tarragona Spain; 12 Departament d’Oncología Médica, Institut Català d’Oncologia, Hospital Universitari Doctor Josep Trueta, Girona, Spain

**Keywords:** Breast cancer, Survival, Probability of death, Stage, Age

## Abstract

**Background:**

We provide population-based long-term survival indicators of breast cancer patients by quantifying the observed survival, and the probabilities of death due to breast cancer and to other causes by age and tumor stage at diagnosis.

**Methods:**

We included a total of 10,195 female patients diagnosed before 85 years with invasive primary breast cancer in Girona and Tarragona during the periods 1985–1994 and 1995–2004 and followed-up until December 31st 2014. The survival indicators were estimated at 5, 10, 15 and 20 years of follow-up comparing diagnostic periods.

**Results:**

Comparing diagnostic periods: I) the probability of death due to other causes did not change; II) the 20-year survival for women diagnosed ≤ 49 years increased 13% (1995–2004 = 68%; 1985–1994:55%), whereas their probability of death due to breast cancer decreased at the same pace (1995–2004 = 29%; 1985–1994 = 42%); III) at 10 years of follow-up, decreases in the probabilities of death due to breast cancer across age groups switched from 11 to 17% resulting in a risk of death reduction of 19% after adjusting by stage. During 1995–2004, the stage-specific 10-year probabilities of death due to breast cancer switched from: 3–6% in stage I, 18–20% in stage II, 34–46% in stage III and surpassed 70% in stage IV beyond 5 years after diagnosis.

**Conclusions:**

In our study, women diagnosed with breast cancer had higher long-term probability to die from breast cancer than from other causes. The improvements in treatment and the lead-time bias in detecting cancer in an early stage resulted in a reduction of 19% in the risk of death between diagnostic periods.

**Electronic supplementary material:**

The online version of this article (10.1007/s12094-018-1852-1) contains supplementary material, which is available to authorized users.

## Introduction

Breast cancer (BC) is the most frequent tumor and the first cause of cancer death among European women in the recent years [1]. In Spain, it has been estimated that 28,000 new BC cases were diagnosed in 2015, with an incidence rate (adjusted for the European population) of 88.3 cases per 100,000 women-years, that leads to an intermediate position in Europe [2]. It is estimated that 1 in 9 Spanish women will develop BC throughout their life [3], with a mean age at diagnosis of 60 years, although BC incidence rates have shown a downturn since 2001 among Spanish women over 45 [4].

Improvements in 5-year BC survival have been observed [5–8] in parallel with a decline in the risk of BC mortality specially marked in women under 50 years of age [9]. Since life expectancy can lengthen by several decades in young BC patients [10], to provide long-term survival estimates is called for. To our knowledge, population-based survival studies in Spain have been provided up to 5-year survival estimates [5–7, 11], but not beyond this follow-up. It is of crucial interest the assessment of the most recent survival estimates, since several international studies have suggested a non-decreasing excess mortality beyond 10 years after BC diagnosis [12–18].

In a previous study, we provided long-term estimates of the survival probabilities of BC patients diagnosed before 50 years by stage and period of diagnosis using a small cohort (*N* = 998) from the population-based cancer registry of Girona [7]. In the present study, we provide survival indicators of BC patients based on a larger cohort of *N* = 10,195 women from two population-based cancer registries, Girona and Tarragona, taking into account the age and BC stage and period of diagnosis. Our aim is in quantifying the contribution of BC to overall mortality, assessing the long-term crude probabilities of death, by age and stage, due to BC and other causes among patients diagnosed before 84 years.

## Materials and methods

### Data

BC data was obtained from the population-based cancer registries of Girona and Tarragona, which cover a population of 771,854 women (2011 Catalonia census) [19]. Each woman with BC in these provinces has been followed-up to December 31st, 2014. In addition to the active and passive follow-up via hospitals, two passive follow-ups were performed using record linkage: one linking BC data with the Catalonian Mortality Registry (which covers the four Catalan provinces, Girona, Tarragona, Lleida and Barcelona) and another linking data with the National Death Index of the Spanish Ministry of Health. The patients not found to be dead at the end of follow-up were considered as censored.

We included a total of *N* = 10,195 female patients aged 15–84 years and diagnosed with invasive primary BC (codes 174 and C50 of the 9th and 10th editions of the International Classification of Diseases, ICD-9 and ICD-10, respectively) during the time periods 1985–1994 (*N* = 4211) and 1995–2004 (*N* = 5984). From these, data on the stage at diagnosis was extracted through medical records review. Variables considered for the analysis were age, partitioned into ≤ 49, (49–59), (59–74) and (74–84), and stage at diagnosis. Stage classification was based on the TNM classification system unified to the 5th edition of the American Joint Committee on cancer staging manual [20] to compare the survival indicators by stage between 1985–1994 and 1995–2004. Patients were classified as stage I, II, III and IV when staging was available at the moment of diagnosis, and missing stage otherwise.

### Statistical analyses

We calculated the observed survival (OS), and the probabilities of death due to BC (PBC) and other causes (POC) by age and stage at diagnosis at 5, 10, 15 and 20 years of follow-up and we compared these three probabilities between time periods at diagnosis 1985–1994 and 1995–2004 and across age groups. Since life expectancy among Catalan women is 90 years [19], we did not estimate these probabilities beyond 5 years of follow-up for patients aged between 74 and 84 years at diagnosis. In the same line, we did not estimate these probabilities beyond 15 years of follow-up for patients aged between 59 and 74 years at diagnosis.

In brief, up to year *T*, *P*(*t* ≤ *T*) is the cumulative probability to die from any cause in the cohort, whereas OS(*T* > *t*) is the cumulative probability to survive in the cohort beyond the year *T*, where OS(*T* > *t*) + *P*(*t* ≤ *T*) = 1. The *P*(*t* ≤ *T*) can be estimated as a sum of two probabilities: *PBC*(*t* ≤ *T*) is the estimated cumulative probability to die due to the disease under study, BC in our case, and POC(*t* ≤ *T*), the estimated cumulative probability to die due to causes other than the cancer of interest [21]. These probabilities can be estimated making use of the excess hazard of death [22, 23] under a competing risk’s approach to survival, as described in a previous study [7]. This approach allows providing estimates of PBC and POC without knowing the exact cause of death [7, 22, 23], where it is only needed to know if the patient dies or not because of any cause at the end of study. Therefore, since OS(*t* > *T*) + PBC(*t* ≤ *T*) + POC(*t* ≤ *T*) = 1, we will present these probabilities depicted in a survival graph in the results section. We derived their corresponding 2.5, and 97.5% percentiles using the R-library relsurv [23].

Since the closing date of follow-up was December 31^st^, 2014, for patients diagnosed during 1995–2004, we estimated the probabilities of interest beyond 10 years of follow-up using different cohorts, as performed in the previous study [7]. Therefore, 11-year follow-up was estimated using the patients diagnosed during the period 1995–2003, whereas 12-year follow-up was estimated using the patients diagnosed during the period 1995–2002 and so on up to 19 years. We used the cohort of patients diagnosed in 1994 to estimate these probabilities at 20-year follow-up.

First we assessed up to 20-year survival estimates between periods of diagnosis across age groups. Second, we compared 10-year OS between 1985–1994 and 1995–2004 across stage and age groups. A Cox proportional hazards model was used to assess hazard ratios of all-cause mortality by age, stage and period of diagnosis. Finally, since stage information was available in 86.2% of the patients diagnosed during 1995–2004, 10-year estimates of the probabilities of interest provide the most recent long-term survival indicators by age and stage in Girona and Tarragona.

The Supplementary material file presents additional tables, figures and an extension of the statistical methods.

## Results

### Comparison of long-term survival between 1985–1994 and 1995–2004 by age groups

**Fig. 1:**
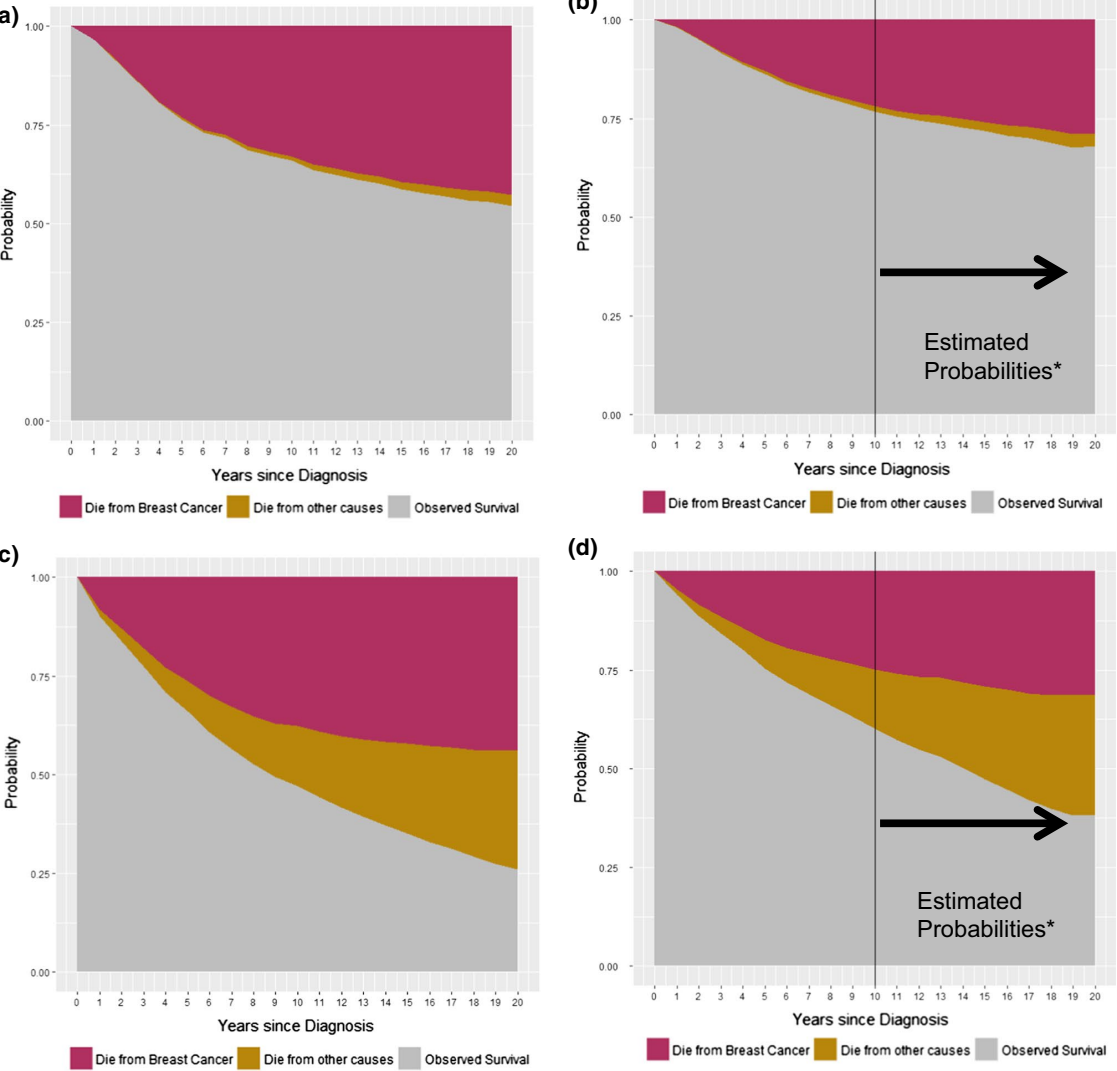
Long-term survival of breast cancer patients diagnosed in Girona and Tarragona before 85 years of age: **a** diagnosed ≤ 49 years during 1985–1994; **b** diagnosed ≤ 49 years during 1995–2004; **c** diagnosed > 49 years during 1985–1994; **d** diagnosed > 49 years during 1995–2004. *Since complete follow-up until December 31st, 2014 for patients diagnosed between 1995–2004 was 10 years, follow-up beyond 10 years was estimated using the cohorts diagnosed between 1995–1993 (11-year probabilities), 1995–1992 (12-year probabilities) and so on

Comparing the time periods 1985–1994 and 1995–2004, there were no significant differences in the age distribution of the patients considered in the cancer registries, see Table 1. Table S1 in the supplementary material presents survival indicators and crude probabilities of death by period of diagnosis across specific age groups. Figure 1 depicts the PBC, POC and the OS comparing survival indicators by period of diagnosis in women ≤ 49 and > 49. This figure shows the improvement in OS in parallel with the reduction in the PBC, whereas POC did not change between diagnostic periods. Worth noting is that, among women ≤ 49, 20-year OS for women was 55% (PBC = 42%) if they were diagnosed during 1985–1994, whereas based on our estimations, it could reach 68% (PBC = 29%) if they were diagnosed during 1995–2004.

**Table 1:** Age-group distribution of the breast cancer patients included in the study during the time periods 1985–1994 and 1995–2004

Age	1985–1994		1995–2004	
	*N*	(%)	*N*	(%)
0–49	1110	26.4	1561	26.1
50–59	844	20.0	1307	21.8
60–74	1564	37.1	2074	34.7
75–84	693	16.5	1042	17.4
TOTAL	4211	100	5984	100

**Fig. 2:**
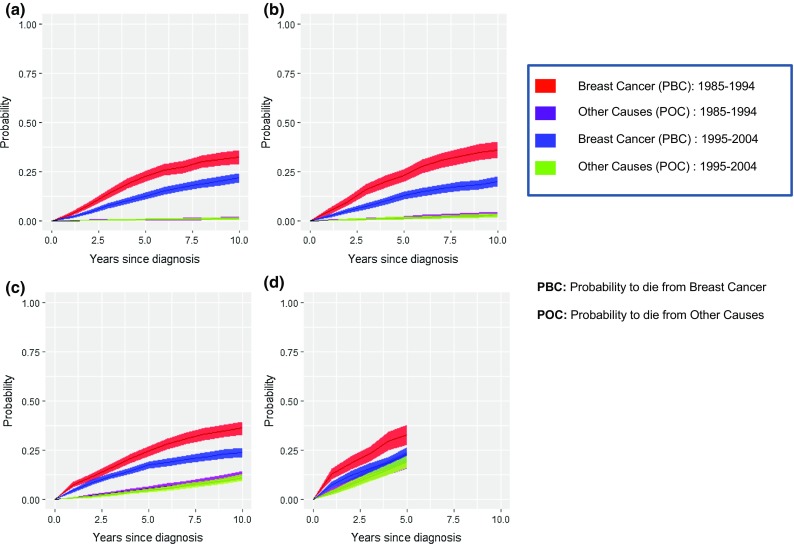
Comparison of the 10-year probabilities of death due to breast cancer and other causes between diagnostic periods 1985–1994 and 1995–2004 by age groups: **a** ≤ 49 years; **b** (50–59) years; **c** (60–74) years; **d** (75–84) years. Shaded bands represent 95% confidence intervals

Comparing 10-year probabilities of death due to BC and OC by period of diagnosis and age group (Fig. 2), differences in the PBC between 1985–1994 and 1995–2004 were 11% among women ≤ 49 (1985–1994: 33%;1995–2004: 22%), 17% among women (50–59) (1985–1994: 37%; 1995–2004: 20%), and 14% among women (60–74) (1985–1994: 37%; 1995–2004:23%), and 10% among women aged (74–84). PBC surpassed POC in all age groups except in the oldest age group, where confidence intervals of POC overlap those of PBC beyond 5 years of follow-up. Beyond 10 years of follow-up (Table S1), the largest differences in PBC between periods could have reached 12% among women aged (59–74) at 15 years (PBC 41% in 1985–1994; 29% in 1995–2004) and 20% among women aged (49–59) at 20 years (48% in 1985–1994; 28% in 1995–2004). Differences in the OS between diagnostic periods are similar to those found in the PBC.

### Comparison of observed survival by age–stage between diagnostic periods

Table 2 presents the stage distribution across age groups by period of diagnosis. Stage was available in 20.6% (*N* = 867) of the patients diagnosed during the period 1985–1994 and in 86.2% (*N* = 5160) in those patients diagnosed during 1995–2004. Among patients with staging information in 1985–1994, there was a higher percentage of patients diagnosed in Stage I during 1995–2004 (34.6%) than during 1985–1994 (26.5%) and a higher percentage of patients diagnosed in stage IV (13.3 vs 6.8%) than during 1995–2004 (6.8%). Differences in the mean age at diagnosis were also assessed and not found to be statistically significant. We also compared 10-year OS between time periods across stages: I) hazard rates in periods 1985–1994 and 1995–2004 were of similar magnitude by age and stage (Table 3), but showing that patients diagnosed during 1995–2004 had 19% reduction in risk of death compared to those diagnosed in 1985–2004; II) we found significant differences in survival between periods in stage I patients (Supplementary material: Figure S1 Wilcoxon test *p* < 0.001; Table S2 10-year OS in 1985–1994: 85%; 1995–2004: 89%) and in stage IV (Wilcoxon test *p* < 0.05; 10-year OS in 1985–1994: 2%; 1995–2004: 7%).

**Table 2:** Stage-distribution and mean age at diagnosis within stage between periods 1985–1994 and 1995–1994

Stage	1985–1994 (*N*) (%) (%*)	Mean age	1995–2004 (*N*) (%) (%**)	Mean age	Differences stage (difference) (95% CI)
I	230 (5.5) (26.5)	56.5	1783 (29.8) (34.6)	58.6	− 8.1*** (− 11.2; − 4.1)
II	392 (9.3) (45.2)	55.6	2300 (38.4) (44.6)	58.9	0.6 (− 3.1; 4.1)
III	130 (3.1) (15.0)	56.8	724 (12.1) (14.0)	59.6	1.0 (− 1.6; 3.5)
IV	115 (2.7) (13.3)	61.6	353 (5.9) (6.8)	63.4	6.46*** (3.9; 8.9)
Total with stage (*N*)	867 (20.6) (100)	56.8	5160 (86.2) (100)	59.2	–
Unknown stage	3344 (79.4) (–)	60.5	824 (13.8) (–)	63.9	–
Total	4211 (100) (–)	59.8	5984 (100) (–)	59.9	–

%, Percentage with respect to the total in each time period

%*, Percentage with respect to *N* = 867 cases with stage confirmed during 1985–1994

%**, Percentage with respect to *N* = 5160 cases with stage confirmed during 1995–2004

*Mean age* mean age at diagnosis

*Differences stage* differences in the percentage of patients in Stage

*95% CI* 95% confidence interval of the difference

***95% confidence interval of the difference does not include 0

**Table 3:** All-cause mortality adjusted by age and stage using a Cox model

	1985–1994			1995–2004				1985–2004		
	HR	95% CI		HR	95% CI			HR	95% CI	
≤ 49	Ref.			Ref.				Ref.		
(50–59)	1.17	1.01	1.35	1.06	0.91	1.06		1.12	1.01	1.24
(60–74)	1.52	1.34	1.72	1.54	1.36	1.54		1.54	1.41	1.68
(75–84)	3.22	2.82	3.68	3.06	2.7	3.06		3.17	2.9	3.48
Stage										
I	Ref.			Ref.				Ref.		
II	1.72	1.22	2.43	2.22	1.93	2.22		2.09	1.84	2.37
III	3.83	2.63	5.56	4.36	3.74	4.36		4.09	3.55	4.72
IV	13.91	9.77	19.78	17.18	14.62	17.18		15.10	13.07	17.45
Missing	3.22	2.39	4.35	3.65	3.14	3.65		3.48	2.98	4.06
							Period			
							85–94	Ref		
							95–04	0.81	0.71	0.91

*Period* period of diagnosis, *HR* hazard ratio, *95% CI* 95% confidence interval, *Ref* reference category

### Age-stage observed survival and crude probabilities of death for patients diagnosed during 1995–2004

Table 4 presents the 5-year and 10-year OS and the crude probabilities of death for the cohort of patients diagnosed during 1995–2004 by age and stage at diagnosis. Differences in 5-year OS switched from 7 to 11% comparing stages I and II, and from 13 to 22% comparing stages II and III. We note that the PBC switched from 1 to 3% in stage I, from 9 to 12% in stage II and from 23 to 34% in stage III. The PBC in stage IV patients was estimated to surpass 70% in all age groups, since these patients showed that less than 29% and less than 20% could survive more than 5 years among those diagnosed ≤ 49 years and beyond 49 years, respectively. These figures must be interpreted with caution due to the small sample size (less than 30 patients at risk).

**Table 4:** Age-specific 5- and 10-year observed survival and crude probabilities of death due to cancer and to other causes among women diagnosed with breast cancer in Girona and Tarragona during the period 1995–2004: results stratified by stage at diagnosis

Age	Stage I				Stage II				Stage III	
	(*N*)	OS (%) (95% CI)	PBC (%) (95% CI)	POC (%) (95% CI)	(*N*)	OS (%) (95% CI)	PBC (%) (95% CI)	POC (%) (95% CI)	(*N*)	OS (%) (95% CI)
(a) 5-year survival										
≤ 49	427	97 (96; 98)	3 (1; 5)	0 (–)	594	90 (88; 92)	10 (7; 11)	0 (–)	166	77 (71; 83)
(49–59)	451	98 (97; 99)	1 (0; 3)	1 (0; 3)	448	88 (86; 90)	11 (9; 13)	1 (0; 3)	106	74 (67; 81)
(59–74)	639	94 (92; 95)	1 (0; 3)	5 (3; 7)	620	83 (81; 85)	12 (10; 14)	5 (3; 7)	132	65 (59; 72)
(75–84)	147	81 (75; 87)	2 (0; 6)	17 (12; 22)	261	70 (65; 75)	9 (5; 14)	21 (16; 25)	75	48 (40; 56)
(b) 10-year survival										
≤ 49	403	92 (89; 94)	6 (4; 8)	2 (0; 4)	520	79 (76; 82)	20 (17; 23)	1 (0; 3)	142	65 (59; 71)
(49–59)	431	94 (92; 96)	3 (1; 5)	3 (1; 5)	400	79 (75; 82)	18 (15; 21)	3 (1; 4)	83	57 (49; 66)
(59–74)	579	85 (83; 88)	3 (1; 5)	12 (10; 14)	503	67 (64; 71)	20 (18; 22)	12 (9; 13)	90	44 (38; 52)
(75–84)	–	–	–	–	–	–	–	–	–	–

Age	Stage III		Stage IV*				Stage missing			
	PBC (%) (95% CI)	POC (%) (95% CI)	(*N*)	OS (%) (95% CI)	PBC (%) (95% CI)	POC (%) (95% CI)	(*N*)	OS (%) (95% CI)	PBC (%) (95% CI)	POC (%) (95% CI)
(a) 5-year survival										
≤ 49	23 (17; 29)	0 (–)	19	29 (20; 42)	70 (58; 82)	0 (–)	129	81 (75; 87)	19 (13; 25)	0 (–)
(49–59)	25 (18; 32)	1 (0; 6)	10	19 (11; 33)	80 (75; 90)	1 (0; 12)	95	75 (65; 81)	24 (18; 34)	1 (0; 6)
(59–74)	30 (24; 36)	5 (0; 9)	21	13 (9; 20)	85 (80; 92)	2 (0; 12)	190	67 (61; 72)	28 (22; 34)	5 (0; 11)
(75–84)	34 (26; 42)	18 (11; 26)	11	12 (6; 21)	82 (77; 91)	6 (0; 18)	105	43 (37; 49)	38 (32; 44)	19 (13; 25)
(b) 10-year survival										
≤ 49	34 (28; 40)	1 (0; 4)	8	10 (5; 21)	88 (81; 97)	2 (0; 12)	112	71 (64; 78)	28 (21; 35)	1 (0; 4)
(49–59)	41 (33; 49)	2 (0; 6)	3	6 (2; 15)	90 (28; 44)	4 (0; 14)	82	62 (54; 71)	36 (28; 44)	3 (0; 6)
(59–74)	46 (40; 52)	10 (4; 15)	10	6 (3; 13)	91 (85; 98)	3 (0; 17)	153	54 (48; 60)	35 (29; 41)	11 (5; 16)
(75–84)	–	–	–	–	–	–	–	–	–	–

*95% CI* 95% confidence interval, *PBC* crude probability of death due to BC, *POC* Crude probability of death due to other causes

*N* patients at risk at follow-up interval; *OS* observed survival, *95% CI* 95% confidence interval, *PBC* crude probability of death due to cancer

*POC* Crude probability of death due to other causes, Stage IV* probabilities must be interpreted with caution due to small cohort size

Similar gradient of differences was found in 10-year OS: 13–18% comparing stages I and II, and 14–23% comparing stages II and III. The 10-year PBC in 1995–2004 switched from 3% (ages (49–59]) to 6% (≤ 49) in stage I, 18% (49–59) to 20% in stage II and from 34% (≤ 49) to 46% (59–74) in stage III. In stage IV patients, PBC surpassed 88% in all age groups and a very small sample size (less than 10 patients) could reach 10-year follow-up.

## Discussion

We have estimated that risk of death among BC patients was reduced by 19% in 1995–2004 compared to 1985–1994 and the improvement in 10-year survival between the two periods of diagnosis was mainly due to the decrease in the PBC, since the POC did not change between these time periods. We have estimated that the difference in the PBC between diagnostic periods may continue or even increase beyond 10 years of follow-up among women diagnosed before 60 years. These survival improvements between periods of diagnoses could be due to better survival prospects for stage I and stage IV patients in 1995–2004 compared to 1985–1994. The most recent estimates of 10-year PBC among BC patients could switch from 3 to 6% in stage I, 18 to 20% in stage II and 34 to 46% in stage III, whereas PBC surpasses 70% in stage IV beyond 5 years of diagnoses.

Our approach has several strengths, in comparison with previous survival studies. First, we provide estimates of the crude probabilities of death due to BC and other causes using population-based cancer registry data from a Spanish cohort of BC patients, and, to date, this is the first study of this type carried out in Spain. These probabilities can be used as indicators to assess the improvement in overall OS and in quantifying the contribution of the disease to overall mortality [22]. Our approach makes a major difference with relative survival. Relative survival is used as population-based cancer survival indicator and as estimator for the net survival, but relative survival is not a survival probability measure [7, 21, 22]. Second, we provided the most recent estimates of the 10-year figures of these probabilities, where our study had 86.2% completeness of this information in 1995–2004.

It is worth noting the strength limitation related with staging classification between diagnostic periods in our study, which was based on the 5th edition of the AJCC staging manual [20]. Since stage migration due to different classifications could artifactually inflate cancer survival rates by shifting patients with better prognosis group into a worse prognosis group [24], we used unified staging classification to minimize this bias. However, there was a limitation related with survival outcomes in the classification of patients making use of the AJCC fifth edition, since differences in survival among BC cases classified into stage IV could exist but not detected using this classification. The sixth edition of the AJCC has further amended the staging classification of patients with supraclavicular metastases at diagnosis to include them into the IIIC category [25], since evidence suggests that these patients had similar outcomes than stage IIIB patients and even better outcomes than patients with visceral stage IV disease [26]. There are other limitations to be noted in the survival comparison between periods of diagnosis. Survival estimates beyond 10 year for patients diagnosed during 1995–2004 were estimated using different subcohorts of patients during this time period. Second, availability of stage information in 1985–1994 was limited to *N* = 867 patients and probability estimates drawn from stage indicators in this time period could not be robust. These could also be biased, since patients with stage information in 1985–1994 had better survival than patients without this information, and this may lead to an underestimation of the survival differences between time periods (Supplementary material: Table S3).

In our study, PBC substantially decreased beyond 1994, but significant excess mortality may remain beyond 10 years after diagnosis. This is in agreement with recent long-term survival studies [7, 18, 27]. Improvements in BC survival between 1985–1994 and 1995–2004 in Spain could be attributable to changes due to BC screening, active in Girona and Tarragona since 1998–1999 [3], management and treatment [6]. In this line, we detected a 19% of risk of death reduction for BC patients diagnosed beyond 1995. Since BC screening is related to lead-time bias, it may increase the number of BC diagnosed at early stages (I) and decrease that number at advanced stages (IV). However, it is also difficult to quantify the contribution of BC screening in the mortality reduction without information about the diagnostic method, clinical or by screening, of tumor [28–30].

The introduction of the screening program may imply a reduction in advanced invasive cancers and then patient’s risk of mastectomy or chemotherapy for breast cancer, or side effects of these, may decrease [31]. In 1994, when most of the screening in Catalonia was opportunistic, rates of screening mammography switched from 27% (women aged 50–64) to 43% (women aged 40–49), whereas in 2004, 61.2% of the invited women participated in the national BC screening program and 75.7% either participated in that program or reported that they had received recent mammograms [30]. Overdiagnosis caused by the screening [30] may also be related with the improvement in survival prospects between periods. The influence of a potential stage migration (Will Rogers phenomenon) is a factor that could have influenced changes in survival by stages but not in the global survival [24]. On the other hand, the contribution in the reduction of BC mortality by treatment improvements is also difficult to quantify, since we found that hazard ratios by age and stage were of similar magnitude between diagnostic periods.

Our probability estimates are based in a competing’ risks modeling for which we attribute the excess risk of death of our patients to BC. This can be seen as the death probabilities that the cancer patients could “expect to have” in the hypothetical scenario where cancer patients have the same death risks as the general population [7]. If we compare the 10-year PBC and POC estimates with the cause-specific proportion of deaths obtained making use of the death certificates (Supplementary material: Table S4) considering all age groups, the difference between PBC and the proportion of deaths due to BC is 1.8% in both periods. In this line, the difference between POC and the proportion of deaths due to other causes is also small: − 1.9% in 1985–1994 and − 1.8% in 1995–2004. In our study, we associated these improvements with the decrease in the PBC between periods, since we did not find significant differences in the 10-year POC between time periods.

Future research will continue updating the follow-up up to 20 years for the most recent cohort. On the other hand, the data available did not allow us to investigate survival by molecular subtype of breast cancer (e.g., luminal A or B, triple negative, HER2), but evidence suggests that the prognostic value of molecular subtype persists when adjusting for age, stage and histological grade, and certain subtypes may metastasized even when the tumor itself is localized [32]. However, this information will be retrieved for the cohort diagnosed beyond 2000, since hormone’s evaluation and HER2 status was not the standard of care in Spain before this year.

## Conclusion

First, our analysis suggests that women diagnosed with BC have higher PBC than POC during the first 10 years after diagnosis. The reduction of 19% in risk of death beyond 1995 was markedly due to the reduction in the PBC, since the POC did not change between periods. Access to BC screening and the improvements in treatment have had a positive impact on survival prospects of BC patients. To continue the improvement in long-term survival rates of BC patients, continued efforts should be pursued in (1) increasing adherence to screening programmes with better quality and in (2) the introduction of therapeutic innovations.

## Electronic supplementary material

Below is the link to the electronic supplementary material.

Supplementary material 1 (DOCX 347 kb)

## Abbreviations

BC: Breast cancer
OS: Observed survival
PBC: Probability of death due to BC
POC: Probability of death due to other causes
AJCC: American Joint Committee on Cancer
