# Supplementary material for: Long-term crude probabilities of death among breast cancer patients by age and stage: a population-based survival study in Northeastern Spain (Girona–Tarragona 1985–2004)
Source: Clin Transl Oncol. 2018 Mar 6;20(10):1252–60. doi: 10.1007/s12094-018-1852-1 (PMC6153860; doi:10.1007/s12094-018-1852-1)

***Supplementary Material:* Long-term crude probabilities of death among breast cancer patients by age and stage: a population-based survival study in Northeastern Spain (Girona-Tarragona 1985-2004)**

Ramon Clèries^1,2^, Alberto Ameijide^3^, Maria Buxó^4^, José Miguel Martínez^5^, Rafael Marcos-Gragera^6,7,8^, Maria-Loreto Vilardell^6,7^, Marià Carulla^3^, Yutaka Yasui^9^, Mireia Vilardell^10^, Josep A. Espinàs^1^, Josep M. Borràs^1,2^, Jaume Galceran^3,11^, Àngel Izquierdo^6,7,12^

**e-mail:** r.cleries@iconcologia.net

1. Pla Director d’Oncologia (GENCAT). IDIBELL, Hospital Duran i Reynals, Gran Via 199-203 1ª planta. L’Hospitalet de Llobregat, 08908 Barcelona, Spain.

2. Departament de Ciències Clíniques. Universitat de Barcelona, Campus de Bellvitge. L’Hospitalet de Llobregat, Barcelona, Spain

*Other institutions*

3. Registre de Càncer de Tarragona. Fundació Lliga per a la Investigació i Prevenció del Càncer (FUNCA), – IISPV. Reus, Tarragona, Spain.

4. Institut d’Investigació Biomèdica de Girona, IDIBGI. C/Dr.Castany s/n. Edifici M2. Parc Hospitalari Martí i Julià. 17190 Salt, Spain.

5. MC MUTUAL. Departamento de Investigación y Análisis de Prestaciones. C/Provenza, 321. 08037. Barcelona, Spain.

6 Unitat d’Epidemiologia i Registre del Càncer de Girona (UERGG). Institut d’Investigació Biomèdica Girona Josep Trueta (IDIBGI). Girona, Spain.

7 Institut Català d’Oncologia (ICO). Girona, Spain.

8 Departament d’Infermeria, Universitat de Girona (UdG). Girona, Spain.

9. Department of Epidemiology and Cancer Control, St. Jude Children's Research Hospital, Memphis, Tennessee, USA 38105.

10. Sección de Estadística del Departamento de Genética, Microbiología y Estadística de la Facultad de Biología. Universidad de Barcelona 08028.

11. Departament de Medicina i Cirurgia. Universitat Rovira i Virgili. Reus, Tarragona, Spain.

12. Departament d’Oncología Médica, Institut Català d’Oncologia, Hospital Universitari Doctor Josep Trueta, . Girona –, Spain

*Data analysis*

*Modeling the crude probabilities of death due to breast cancer and other causes*

Survival analyses were carried out by modeling the excess hazard of death, which provides a measure of the number of deaths that exceed expectations for the study population. Under the additive excess hazard of mortality [21] model for survival analysis, the observed hazard of death for any cause $\lambda_{O}(t)$at annual interval $t$for persons diagnosed with cancer has been modeled as the sum of the expected hazard of death , this last calculated using the all-cause mortality tables of Girona and Tarragona, and the excess hazard of death due to a diagnosis of cancer$\lambda_{X}(t)$, therefore,

$\lambda_{O}(t)=\lambda_{X}(t)+\lambda_{E}(t)$, $t\in\{1,..,20\}$ (1).

The probability to die from any cause up to follow-up time T, $P(t\leq T)$ was calculated as a function of the observed survival, $P(t\leq T)=\int_{o}^{T} OS(t)\lambda_{O}(t)dt$ [21]. Taking into account all-cause mortality as a whole, making use of (1) we can divide $P(t\leq T)$ into two additive probabilities [21]

$P(t\leq T)=\int_{o}^{T} OS(t)[\lambda_{x}(t)+\lambda_{E}(t)]dt=\int_{o}^{T} OS(t)\lambda_{X}(t)dt+\int_{o}^{T} OS(t)\lambda_{E}(t)dt=PBC(t\leq T)+POC(t\leq T)$ (2),

where $PBC(t\leq T)$is the estimated cumulative probability to die due to the disease under study, BC in our case, up to T, whereas $POC(t\leq T)$is the estimated cumulative probability to die due to causes other than BC [21]. Note that $S(t>T)+P(t\leq T)=S(t>T)+PBC(t\leq T)+POC(t\leq T)=1$. In the results section, we will present these probabilities and the OS depicted in a survival graph.

We provided point estimate and the 95% bootstrap confidence intervals for the OS, PBC and POC using 1000 replicates of the original sample cohort, deriving these probabilities based on the hazard functions[22]. The procedure generates a random sample with replacement (SC) from the original cohort, where the sample size of SC is the same as the original cohort. Then at each annual interval $t|t=1,...,20$ calculates $\lambda_{O}(t)$, and $\lambda_{X}(t)$. From these hazards calculate the probabilities in (2), and keep the OS, PBC and POC point estimates. Repeat this resampling process B=1000 times, and from these 1000 samples of these probabilities one can derive their corresponding 2.5%, and 97.5% percentiles. The statistical software R was used to estimate these probabilities using the library relsurv [23].

|  | 1. ***Patients diagnosed between 1985-1994*** | | | | | | | | | | | | | | | | | |  | |
| --- | --- | --- | --- | --- | --- | --- | --- | --- | --- | --- | --- | --- | --- | --- | --- | --- | --- | --- | --- | --- |
|  |  |  | ***5-year*** |  |  | ***10-year*** | | |  |  | ***15-year*** | | |  |  | ***20-year*** | | | |  | |
| *Ages* | ***(N)*** | ***OS(%)***  ***(95%CI)*** | ***PBC(%)***  ***(95%CI)*** | ***POC(%)***  ***(95%CI)*** | ***(N)*** | ***OS(%)***  ***(95%CI)*** | ***PBC(%)***  ***(95%CI)*** | ***POC(%)***  ***(95%CI)*** |  | ***(N)*** | ***OS(%)***  ***(95%CI)*** | ***PBC(%)***  ***(95%CI)*** | ***POC(%)***  ***(95%CI)*** |  | ***(N)*** | ***OS(%)***  ***(95%CI)*** | ***PBC(%)***  ***(95%CI)*** | ***POC(%)***  ***(95%CI)*** | |  | |  |
| *All ages** | *2908* | *68*  *(67;70)* | *25*  *(22;27)* | *7*  *(3;11)* | *2195* | *52*  *(50;54)* | *35*  *(33;37)* | *13*  *(11,15)* |  | *1742* | *41*  *(39;43)* | *39*  *(37;41)* | *20*  *(18;22)* |  | *1305* | *33*  *(31;35)* | *42*  *(40;44)* | *25*  *(22;27)* | |  | |  |
| ≤*49* | *851* | *76*  *(74;78)* | *23*  *(20;25)* | *1*  *(0;2)* | *733* | *66*  *(63;68)* | *33*  *(29;36)* | *1*  *(0;2)* |  | *651* | *58*  *(55;62)* | *40*  *(36;44)* | *2*  *(0;3)* |  | *561* | *55*  *(52;58)* | *42*  *(38;46)* | *3*  *(1; 5)* | |  | |  |
| *(49-59]* | *637* | *75*  *(72;78)* | *23*  *(19;26)* | *2*  *(0;4)* | *509* | *60*  *(57;64)* | *37*  *(32;41)* | *3*  *(1;5)* |  | *441* | *52*  *(49;55)* | *43*  *(38;47)* | *5*  *(3;8)* |  | *354* | *44*  *(41;48)* | *48*  *(42;52)* | *8*  *(5;11)* | |  | |  |
| *(59-74]* | *1096* | *70*  *(67;72)* | *25*  *(20;29)* | *5*  *(0;10)* | *802* | *51*  *(49;54)* | *37*  *(33;40)* | *12*  *(10;14)* |  | *591* | *38*  *(35;40)* | *41*  *(37;45)* | *21*  *(17;23)* |  | *372* | *-* | *-* | *-* | |  | |  |
| *(74-84]* | *324* | *47*  *(43;51)* | *33*  *(29;35)* | *20*  *(18;22)* | *-* | *-* | *-* | *-* |  | *-* | *-* | *-* | *-* |  | *-* | *-* | *-* | *-* | |  | |  |
|  | 1. ***Patients diagnosed between 1995-2004*** | | | | | | | | | | | | | | | | | | |
|  |  |  | ***5-year*** |  |  | ***10-year*** | | |  | ***15-year**** | | | |  | ***20-year***** | | | | |
| *Ages* | ***(N)*** | ***OS(%)***  ***(95%CI)*** | ***PBC(%)***  ***(95%CI)*** | ***POC(%)***  ***(95%CI)*** | ***(N)*** | ***OS(%)***  ***(95%CI)*** | ***PBC(%)***  ***(95%CI)*** | ***POC(%)***  ***(95%CI)*** |  | ***(N*)*** | ***OS(%)***  ***(95%CI)*** | ***PBC(%)***  ***(95%CI)*** | ***POC(%)***  ***(95%CI)*** |  | ***(N**)*** | ***OS(%)***  ***(95%CI)*** | ***PBC(%)***  ***(95%CI)*** | ***POC(%)***  ***(95%CI)*** | |
| *All ages** | *4637* | *78*  *(77;79)* | *15*  *(14;16)* | *7*  *(5;6)* | *3812* | *64*  *(63;65)* | *23*  *(21;24)* | *13*  *(11;14)* |  | *1358* | *54*  *(52;56)* | *28*  *(26;30)* | *18*  *(16;20)* |  | *224* | *46*  *(41;50)* | *30*  *(26;34)* | *24*  *(20;28)* | |
| ≤*49* | *1335* | *87*  *(85;89)* | *13*  *(11;15)* | *0*  *(0;2)* | *1183* | *77*  *(75;79)* | *22*  *(19;24)* | *1*  *(0;2)* |  | *495* | *73*  *(70;75)* | *25*  *(21;29)* | *2*  *(0;4)* |  | *89* | *68*  *(65;71)* | *29*  *(19;38)* | *3*  *(0;7)* | |
| *(49-59]* | *1110* | *86*  *(84;88)* | *13*  *(10;16)* | *1*  *(0;3)* | *997* | *77*  *(75;79)* | *20*  *(17;22)* | *3*  *(1;4)* |  | *359* | *70*  *(67;73)* | *25*  *(21;29)* | *5*  *(2;7)* |  | *74* | *64*  *(60;68)* | *28*  *(18;38)* | *8*  *(2;14)* | |
| *(59-74]* | *1599* | *78*  *(76;80)* | *17*  *(15;19)* | *5*  *(3;7)* | *1331* | *65*  *(62;0.67)* | *23*  *(22;26)* | *12*  *(9;13)* |  | *453* | *50*  *(48;53)* | *29*  *(25;33)* | *21*  *(16;24)* |  | *67* | *-* | *-* | *-* | |
| *(74-84]* | *497* | *58*  *(55;61)* | *23*  *(20;26)* | *19*  *(16;22)* | *-* | *-* | *-* | *-* |  | *-* | *-* | *-* | *-* |  | *-* | *-* | *-* | *-* | |

**(N):** Number at risk; **OS:** Observed Survival; **(N*):** Number at risk using the cohort of patients diagnosed between 1995-1999; **(N**):** Number at risk using the cohort of patients diagnosed in 1995;

**95% CI:** 95% Confidence Interval; **PBC:** crude probability of death due to BC; **POC:** Crude probability of death due to other causes; **All*:** includes all ages

**Table S1.** Age-specific observed survival and crude probabilities of death due to cancer and to other causes among women diagnosed with breast cancer in Girona and Tarragona during the periods a) 1985-1994 and b) 1995-2004.

|  |  |  |  |  |  |  |  |  |  |  |  |  |  |  |
| --- | --- | --- | --- | --- | --- | --- | --- | --- | --- | --- | --- | --- | --- | --- |
|  | ***1985-1994*** | | | | | |  | ***1995-2004*** | | | | | |  |
|  | **Cohort(N)** | **Years** | **Risk** | **OS(%)** | **LL** | **UL** |  | **Cohort (N)** | **Years** | **Risk** | **OS (%)** | **LL** | **UL** |  |
|  | 772 | 5 | 590 | 76 | 73 | 79 |  | 4362 | 5 | 3644 | 84 | 83 | 85 |  |
|  |  | 10 | 501 | 64 | 60 | 68 |  |  | 10 | 3170 | 73 | 72 | 75 |  |

|  | ***STAGE I: 1985-1994*** | | | | | |  | ***STAGE I: 1995-2004*** | | | | | |
| --- | --- | --- | --- | --- | --- | --- | --- | --- | --- | --- | --- | --- | --- |
|  | **Cohort(N)** | **Years** | **Risk** | **OS(%)** | **LL** | **UL** |  | **Cohort (N)** | **Years** | **Risk** | **OS (%)** | **LL** | **UL** |
|  | 210 | 5 | 200 | 95 | 92 | 98 |  | 1600 | 5 | 1519 | 96 | 95 | 97 |
|  |  | 10 | 178 | 85 | 80 | 89 |  |  | 10 | 1413 | 89 | 88 | 91 |

|  | ***STAGE II: 1985-1994*** | | | | | |  | ***STAGE II: 1995-2004*** | | | | | |
| --- | --- | --- | --- | --- | --- | --- | --- | --- | --- | --- | --- | --- | --- |
|  | **Cohort(N)** | **Years** | **Risk** | **OS(%)** | **LL** | **UL** |  | **Cohort (N)** | **Years** | **Risk** | **OS (%)** | **LL** | **UL** |
|  | 355 | 5 | 303 | 85 | 82 | 89 |  | 1925 | 5 | 1670 | 87 | 85 | 88 |
|  |  | 10 | 265 | 74 | 69 | 79 |  |  | 10 | 1425 | 75 | 73 | 77 |

|  | ***STAGE III: 1985-1994*** | | | | | |  | ***STAGE III: 1995-2004*** | | | | | |
| --- | --- | --- | --- | --- | --- | --- | --- | --- | --- | --- | --- | --- | --- |
|  | **Cohort(N)** | **Years** | **Risk** | **OS(%)** | **LL** | **UL** |  | **Cohort (N)** | **Years** | **Risk** | **OS (%)** | **LL** | **UL** |
|  | 117 | 5 | 74 | 63 | 55 | 73 |  | 566 | 5 | 406 | 72 | 68 | 76 |
|  |  | 10 | 56 | 48 | 39 | 58 |  |  | 10 | 313 | 55 | 51 | 59 |

|  | ***STAGE IV: 1985-1994*** | | | | | |  | ***STAGE IV: 1995-2004*** | | | | | |
| --- | --- | --- | --- | --- | --- | --- | --- | --- | --- | --- | --- | --- | --- |
|  | **Cohort(N)** | **Years** | **Risk** | **OS(%)** | **LL** | **UL** |  | **Cohort (N)** | **Years** | **Risk** | **OS (%)** | **LL** | **UL** |
|  | 90 | 5 | 13 | 14 | 8 | 24 |  | 271 | 5 | 49 | 18 | 14 | 23 |
|  |  | 10 | 2 | 2 | 0 | 8 |  | 772 | 10 | 19 | 7 | 4 | 11 |

**Risk:** Number of patients at risk at follow-up years;**OS:** Observed survival; **LL:** Lower limit of the 95% confidence interval of the OS; **UL:** Upper limit of the 95% confidence interval of the OS;**Cohort(N):** Number of patients at risk at the beginning of study.

**Table S2.** Comparison of 5-year and 10-year observed survival by stage and between periods at diagnosis among patients diagnosed before 75 years with stage available at diagnosis.

|  |  |  |  |  |  |  |  |  |  |  |  |  |  |  |
| --- | --- | --- | --- | --- | --- | --- | --- | --- | --- | --- | --- | --- | --- | --- |
|  | ***MISSING STAGE AT DIAGNOSIS*** | | | | | |  | ***STAGE AVAILABLE AT DIAGNOSIS*** | | | | | |  |
|  | **Cohort(N)** | **Years** | **Risk** | **OS(%)** | **LL** | **UL** |  | **Cohort (N)** | **Years** | **Risk** | **OS (%)** | **LL** | **UL** |  |
|  | 2746 | 5 | 1815 | 66 | 64 | 68 |  | 772 | 5 | 592 | 76 | 73 | 79 |  |
|  |  | 10 | 1547 | 56 | 54 | 58 |  | 772 | 10 | 501 | 64 | 60 | 68 |  |
|  |  |  |  |  |  |  |  |  |  |  |  |  |  |  |

**Risk:** Number of patients at risk at follow-up years;

**OS:** Observed survival; **LL:** Lower limit of the 95% confidence interval of the OS; **UL:** Upper limit of the 95% confidence interval of the OS.

**Cohort(N):** Number of patients at risk at the beginning of study.

**Table S3.** Comparison of survival curves in 1985-1994 between patients diagnosed before 75 years with missing stage at diagnosis with respect to those with stage available at diagnosis.

|  |  |  |  |  |  |  |  |  |  |  |  |  |  |  |  |  |  |  |
| --- | --- | --- | --- | --- | --- | --- | --- | --- | --- | --- | --- | --- | --- | --- | --- | --- | --- | --- |
|  |  |  |  | **1985-1994** | | | | | | | | |  | **1995-2004** | | | |  |
|  |  |  |  | **Follow-up 10-year** | | | |  | **Follow-up 20-year** | | | |  | **Follow-up 10-year** | | | |  |
|  | **Age** | **Cause of death** |  | **N** | **(%)** | **Probability*** | **Dif** |  | **N** | **(%)** | **Probability*** | **Dif** |  | **N** | **(%)** | **Probability*** | **Dif** |  |
|  |  |  |  |  |  |  |  |  |  |  |  |  |  |  |  |  |  |  |
|  | *<=59* | *Breast Cancer* |  | 610 | 31.2 | 34.3 | 3.1 |  | 787 | 40.3 | 44 | 3.7 |  | 553 | 19.3 | 21.4 | 2.1 |  |
|  |  | *Other* |  | 106 | 5.4 | 2.3 | -3.1 |  | 191 | 9.8 | 6.1 | -3.7 |  | 78 | 4.8 | 2.7 | -2.1 |  |
|  |  |  |  |  |  |  |  |  |  |  |  |  |  |  |  |  |  |  |
|  | *60-74* | *Breast Cancer* |  | 530 | 33.8 | 36.6 | 2.8 |  | 627 | 40.1 | 43 | 2.9 |  | 455 | 21.9 | 24.4 | 2.5 |  |
|  |  | *Other* |  | 233 | 14.9 | 12.1 | -2.8 |  | 528 | 33.9 | 31 | -2.9 |  | 289 | 13.9 | 11.4 | -2.5 |  |
|  |  |  |  |  |  |  |  |  |  |  |  |  |  |  |  |  |  |  |
|  | *75-84* | *Breast Cancer* |  | 316 | 45.6 | 43.1 | -2.5 |  | 337 | 48.6 | 45.8 | -2.8 |  | 383 | 36.8 | 33.8 | -3 |  |
|  |  | *Other* |  | 226 | 32.6 | 35.1 | 2.5 |  | 332 | 47.9 | 50.7 | 2.8 |  | 356 | 34.2 | 37.2 | 3 |  |
|  |  |  |  |  |  |  |  |  |  |  |  |  |  |  |  |  |  |  |
|  | All | *Breast Cancer* |  | 1456 | 34.58 | 36.4 | 1.82 |  | 1751 | 41.6 | 43.6 | 2 |  | 1342 | 22.4 | 24.2 | 1.8 |  |
|  |  | *Other* |  | 565 | 13.41 | 11.5 | -1.91 |  | 1051 | 24.9 | 22.9 | -2 |  | 831 | 13.8 | 12 | -1.8 |  |
|  |  |  |  |  |  |  |  |  |  |  |  |  |  |  |  |  |  |  |

**(N):** Number of deaths; **Probability*:** crude probability of death; **Dif.:** difference between crude probability of death and the percentage of death.

**Table S4.** Comparison of the percentage of deaths due to cancer and due to other causes with respect to the crude probabilities of death from breast cancer and from other causes stratified by follow-up during the periods 1985-2004 and 1995-2004.


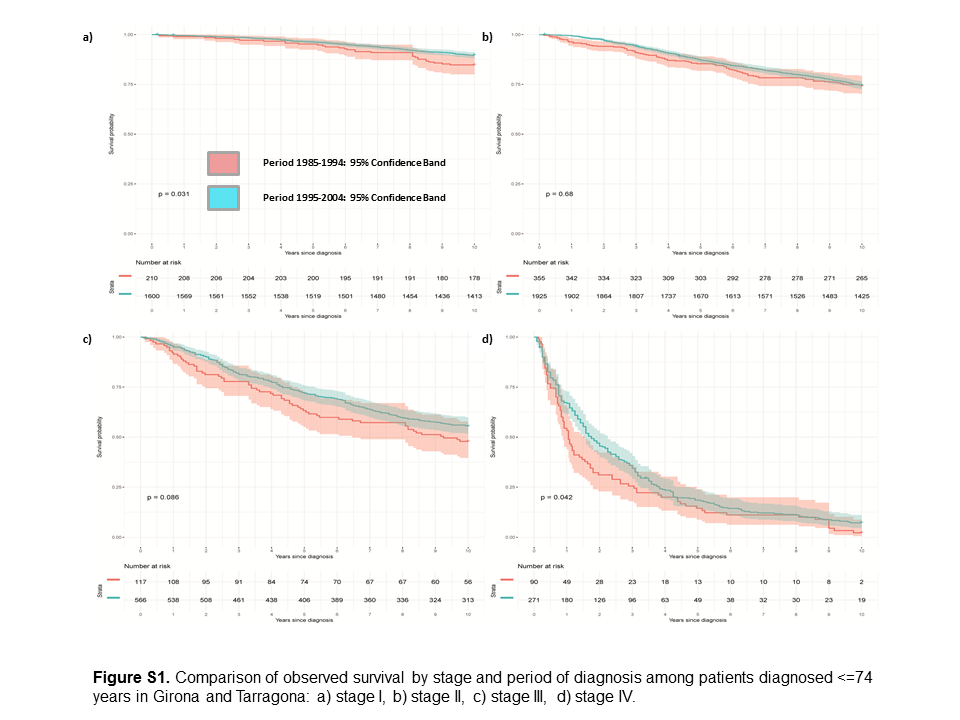

Supplement: Supplementary file 1 — Supplementary material 1 (DOCX 347 kb) [file 12094_2018_1852_MOESM1_ESM.docx]
